# Estimates of healthcare spending for preterm and low-birthweight infants in a commercially insured population: 2008–2016

**DOI:** 10.1038/s41372-020-0635-z

**Published:** 2020-02-26

**Authors:** Andrew L. Beam, Inbar Fried, Nathan Palmer, Denis Agniel, Gabriel Brat, Kathe Fox, Isaac Kohane, Anna Sinaiko, John A. F. Zupancic, Joanne Armstrong

**Affiliations:** 1 Department of Biomedical Informatics, Harvard Medical School, Boston, MA USA; 2 Department of Epidemiology, Harvard T.H. Chan School of Public Health, Boston, MA USA; 3 Department of Newborn Medicine, Brigham and Women’s Hospital, Boston, MA USA; 4 University of North Carolina, School of Medicine, Chapel Hill, NC USA; 5 Department of Surgery, Beth Israel Deaconess Medical Center, Boston, MA USA; 6 Department of Health Policy and Management, Harvard T.H. Chan School of Public Health, Boston, MA USA; 7 Department of Neonatology, Beth Israel Deaconess Medical Center, Boston, MA USA; 8 Department of Women’s Health, Aetna Inc, Boston, MA USA

**Keywords:** Health care economics, Paediatrics

## Abstract

The growth in healthcare spending is an important topic in the United States, and preterm and low-birthweight infants have some of the highest healthcare expenditures of any patient population. We performed a retrospective cohort study of spending in this population using a large, national claims database of commercially insured individuals. A total of 763,566 infants with insurance coverage through Aetna, Inc. for the first 6 months of post-natal life were included, and received approximately $8.4 billion (2016 USD) in healthcare services. Infants with billing codes indicating preterm status (<37 weeks, *n* = 50,511) incurred medical expenditures of $76,153 on average, while low-birthweight status (<2500 g) was associated with average spending of $114,437. Infants born at 24 weeks gestation (*n* = 418) had the highest per infant average expenditures of $603,778. Understanding the drivers of variation in costs within gestational age and birthweight bands is an important target for future studies.

## Introduction

Preterm birth and low birthweight exert significant medical, social, and economic costs on affected families, as well as the United States (US) healthcare system. In 2017, over 372,000 infants in the US were born preterm (age <37 weeks of gestation), representing 9.9% of all live births, and 8.3% of all live births were born at low birthweight (<2500 g) [1]. Preterm birth is the leading cause of neonatal mortality and is a significant cause of both short and long term infant morbidity and disability [2].

The economic impact of preterm birth is also substantial. In one of the most comprehensive studies to date, a 2007 Institute of Medicine (IOM) [2] review estimated that the societal economic burden associated with prematurity in the US was at least $26.2 billion annually in 2005 dollars, or $51,600 per infant born preterm. Among the total economic costs, the IOM estimated the *direct* per capita medical care costs of prematurity in 2005 dollars to be ~$32,300, the majority (85%) of which occurs during the first year of life, compared with $3325 for term infants. Similar estimates have been reported in an analysis of a large cohort of births in California [3] and in a separate analysis of a cohort from the Nationwide Inpatient Sample data [4]. A single-year study of insurance claims from 2013 of 12-month incremental private expenditures estimated a range of $47,000–$78,000 [5] per infant coded as being preterm. A recent study [6] of infants in the California cohort from 2009 to 2011 found that the average newborn costs for infants born preterm was $48,036.

Our study presents an 8-year window into healthcare expenditures for preterm and low-birthweight infants in a commercially insured population and provides a complementary perspective to recent studies [2–4, 6], which have primarily focused on medical production costs for preterm infants. Production costs represent the amount incurred by the hospital to provide a given medical service, whereas an expenditure is the amount billed to the payor. In general, these can be very different quantities and expenditures can vary greatly by payor. Factors that could influence medical expenditures for preterm infants may have changed and vary by the type of population studied, and thus more up to date and comprehensive figures are needed to complete the spending picture for this patient population. These factors include technological advancements in maternal and neonatal care, medical cost inflation, variation in care based on supply sensitive influences [7], differences in reimbursement arrangements between commercial and government insured populations, and health system redesign possibly in response to changes in provider payment such as bundled payments and value based provider contracting. Accurate estimates of the total medical care expenditures of prematurity and low birthweight in the current environment have been identified as a priority for health policy researchers and budget planners [8]. Healthcare spending stratified by gestational age and birthweight is needed to evaluate the potential impacts of medical interventions, research efforts, and policy decisions that aim to reduce the rate of preterm or low-birthweight infants. Moreover, judicious healthcare delivery (often referred to as “choosing wisely” [9, 10]) for preterm infants requires a comprehensive understanding of costs and spending for this population.

## Materials methods

### Data source

The study data were drawn from a national, deidentified administrative database of ~45 million individuals with a commercial insurance plan through Aetna, Inc. from January 2008 to February 2016. This cohort has been used previously to analyze opioid prescribing patterns [11], recurrence patterns in autism [12], and estimates of the heritability of certain conditions using twins [13]. Linkage between maternal and infant records was achieved using a family structure table provided by the insurer. Diagnostic history and patient characteristics were assessed using the codes from the *International Classification of Diseases*, Ninth Revision (ICD-9) and the *International Classification of Diseases*, Tenth Revision (ICD-10) available through claims filed with the insurer. Infants born in California (*n* = 131,171) were excluded because many provider contracts in the state include capitated or other contract arrangements that can decrease the reliability of claims data at the specific member/patient level. This database did not contain any race, ethnicity, or socioeconomic information. The Harvard Medical School Institutional Review Board waived the requirement for approval, as it deemed this analysis of the database to not be human subjects research.

### Cohort definition

**Fig. 1:**
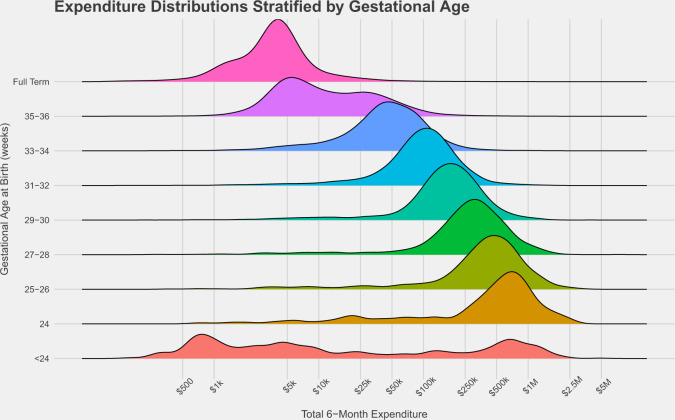
6-month spending distributions, stratified by gestational age. The *y*-axis displays the gestational age group estimated from ICD-9/10 codes at birth and the *x*-axis displays the total 6-month expenditures in dollars.

Our study cohort included all infants who were enrolled in an insurance plan for the first 6 months of life and infants who were enrolled in a plan but died prior to 6 months of age. Gestational age that is “preterm” was determined on the basis of ICD-9 and ICD-10 billing codes (ICD-9: 765.20–765.28, ICD-10: P07.20–P07.39), as was “low birthweight” (ICD-9 765.00–765.18, ICD-10 P07.00–P07.18). Preterm infants with unspecified gestational age codes (ICD-9 = 765.20, ICD-10 P07.20, *n* = 1481) were excluded from analyses that stratified by gestational age. Likewise, low-birthweight infants with unspecified birthweights (ICD-9 = 765.00/765.01, ICD-10 = P07.00/P07.01, *n* = 4825) were excluded from analyses that stratified by birthweight. Infants who did not receive a code for preterm or low birthweight were grouped into “Full Term” and “Normal Birthweight”, respectively. It is known that administrative claims databases may contain errors, including inaccuracies in the billing codes [14, 15]. We investigated how this could affect our cohort by calculating the number of clinically implausible combinations of gestational age and birthweight according to the methodology in Olsen et al. [16] (refer to supplement for the full details on the methodology and comparisons). We found a very low rate (0.27%) of implausible birthweight/gestational age combinations. In addition, among infants coded as preterm, the percentage born in each gestation age band (e.g. 27–28 weeks) closely aligned with external figures [1] provided by the Centers for Disease Control and Prevention.

Occurrence of any of the following adverse events during the first 6 months of life were identified using ICD-9/10 codes: necrotizing enterocolitis (NEC) (ICD-9 777.5, ICD-10 P77.9), retinopathy of prematurity (ROP) (ICD-9 362.20–362.28, ICD-10 H35.10 – H35.16), respiratory distress syndrome (RDS) (ICD-9 769, ICD-10 P22.0), bronchopulmonary dysplasia (BPD) (ICD-9 770.7, ICD-10 P27.1), and neonatal sepsis (ICD-9 771.81, ICD-10 P36).

### Calculation of length of stay and direct medical expenditures

The total infant initial hospital length of stay (LOS) was computed as the total number of hospital days from birth until first discharge date. This calculation includes any inter-hospital transfers following birth and represents the total number of days an infant stayed in the neonatal intensive care unit (NICU) until their home discharge.

Spending estimates were calculated using “allowed” amounts for all medical services received during the first 6 months of life, excluding outpatient pharmacy. Allowed amounts are the total amounts paid to a provider for medical services, including both insurer-paid payments and patient-paid (e.g., co-payments, deductibles) and thus are an accurate estimate of the total amount spent on care from the perspective of private third-party payers on care [17]. Allowed neonatal expenditures in this analysis include: all inpatient and outpatient services incurred over the 6 months following birth including all hospitalization costs (including any readmissions), inpatient medication costs, all inpatient and outpatient professional physician costs, and ancillary services including radiology, laboratory, and respiratory care services. Outpatient pharmacy services were excluded from the analysis because not all individuals in the cohort also had pharmacy coverage under their insurance plan. All expenditures were adjusted to the last year of the study (2016) dollars using the healthcare component of the personal consumption expenditure (PCE) index obtained through the US Federal Reserve Economic Data (FRED) website (https://fred.stlouisfed.org/series/DHLCRG3Q086SBEA). We excluded any infants with a total spend <$500 (*n* = 6972), as they likely represented incomplete or erroneous claims records based on health plan experience.

### Statistical analysis

The distribution of healthcare expenditures and summary statistics, stratified by gestational age and birthweight, were computed and visualized. Smoothed estimates for the spending distributions were computed using a locally estimated scatterplot smoother. Regression analysis to estimate the association of sex, gestational age, birthweight, and adverse event status on spending was performed using a generalized linear model with a logarithmic link function and a gamma distribution [18]. Adverse event status was coded as a binary variable indicating the presence or absence of at least one claim indicating the condition. For each adverse event, we report a “spending multiplier” quantity. Spending multipliers are exponentiated regression coefficients and represent the multiplicative increase in average spending associated with the occurrence of an adverse event on the logarithmic scale, holding all other variables constant. We report spending multipliers from the full model which contained all adverse events, gestational age, birthweight, and sex as variables. All statistical analysis was performed using the R statistical programming language [19].

## Results

### Overview

In total, there were 763,566 infants who met the study inclusion criteria associated with ~$8.4 billion in healthcare spending during the study period. Among these infants, 64,575 (8.5%) were preterm and 45,708 (6.0%) were low birthweight. Using ICD billing codes, we were able to estimate approximate gestational ages (e.g., excluding codes for “unspecified gestational age”) for 50,512 (78%) preterm infants and approximate birthweights for 32,508 (71%) of LBW infants. Among infants born preterm, the percentage of infants born in each gestational age ban in our data closely mirrored the figures provided by the Centers for Disease Control in the 2015 vital statistics [1] (Supplementary Fig. 1). There were 713,253 full-term infants and 727,538 infants born at a normal birthweight.

### Total 6-month expenditures stratified by gestational age and birthweight

**Fig. 2:**
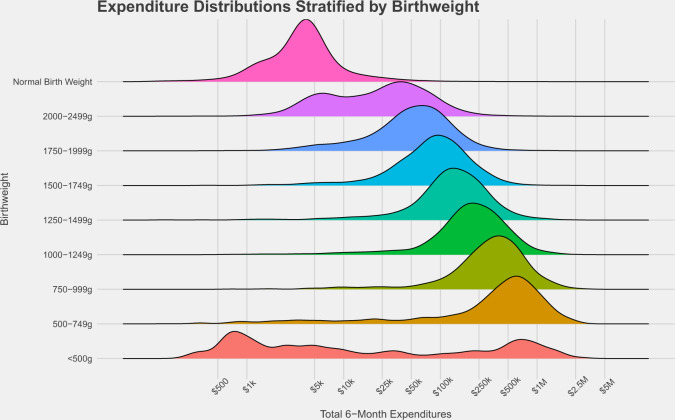
6-month spending distributions, stratified by birthweight. The *y*-axis displays the birthweight group estimated from ICD-9/10 codes at birth and the *x*-axis displays the total 6-month expenditures in dollars.

Table 1 gives an overview of the main results including sample size, average per infant spending, adverse event prevalence, and length of stay. Figure 1 displays a visualization of total 6-month expenditures for each gestational age cohort, and Fig. 2 contains the corresponding figure stratified by birthweight. The average 6-month expenditure for preterm infants was $76,153 (standard deviation = $169,931, median = $26,374, *n* = 50,512) while the 6-month expenditure for infants with low-birthweight status was $114,437 (standard deviation = $460,159, median = $48,906, *n* = 32,508). When stratifying by gestational age, infants born at 24 weeks had the highest average expenditures of $603,778 (standard deviation = $509,165, median = $548,865, *n* = 418). When stratifying by birthweight, infants with a birthweight of 500–749 g experienced the highest expenditures of $537,624 on average (standard deviation = $460,159, median = $467,490, *n* = 1002). In comparison, a full-term infant had an average spending of $6370 (standard deviation = $29,170, median = $3787, *n* = 713,253), and a normal birthweight infant had an average expenditure of $6743 (standard deviation = $30,360, median = $3826, *n* = 727,538).

**Table 1:** Overview of the infants in each gestational age and birthweight cohort.

	Expenditures					Adverse event prevalence					Length of stay		
	*n*	Median	Mean (SD)	Total	Mortality	BPD	RDS	ROP	NEC	SEP	*n*	Mean (SD)	Median
*Infants with documented gestational age*													
Weeks of gestation													
<24	536	$7,318	$242,887 ($458,312)	$130,187,254	60.40%	26.50%	38.80%	21.10%	8.80%	24.60%	524	29 (50)	2
24	418	$548,865	$603,778 ($509,165)	$252,379,413	24.40%	65.30%	89%	59.60%	12.70%	49%	403	80 (59)	92
25–26	984	$418,191	$493,304 ($396,830)	$485,410,893	10.70%	60.40%	91.10%	65.90%	11%	46.20%	947	77 (45)	81
27–28	1357	$291,029	$356,839 ($334,460)	$484,230,274	3.40%	38.80%	89.30%	60.50%	7.70%	31.30%	1306	63 (31)	64
29–30	2182	$173,638	$213,353 ($191,232)	$465,536,989	1.70%	14.80%	79.40%	47.80%	4.30%	25.20%	2106	46 (24)	44
31–32	4562	$100,032	$124,553 ($118,343)	$568,209,519	1%	3.90%	57.80%	19.30%	1.90%	18.50%	4397	29 (14)	28
33–34	11,999	$45,580	$62,994 ($91,189)	$755,860,528	0.60%	0.80%	30.60%	1.80%	0.80%	14.40%	11486	15 (11)	14
35–36	28,474	$9,864	$24,754 ($72,519)	$704,847,939	0.30%	0.20%	10.50%	0.20%	0.10%	5.50%	26648	6 (8)	3
All preterm with specified GA	50,512	$26,374	$76,153 ($169,931)	$3,846,662,810	3%	4.40%	27.90%	8%	1.30%	12.40%	560077	4 (8)	2
Full term	713,253	$3,787	$6,370 ($29,170)	$4,543,558,496	0.10%	0%	0.10%	0%	0%	0%	512260	2 (4)	2
*Infants with documented birthweight*													
Birthweight													
<500 g	393	$6,379	$247,994 ($450,490)	$97,461,782	59.30%	24.20%	37.70%	22.60%	5.10%	21.90%	381	32 (54)	2
500–749 g	1002	$467,490	$537,624 ($460,159)	$538,699,110	22.80%	59.80%	85.90%	58.20%	13.10%	43.40%	970	76 (57)	84
750–999 g	1462	$356,300	$428,791 ($343,145)	$626,892,003	7%	49.20%	89.30%	65.20%	9.40%	37.30%	1424	73 (39)	73
1000–1249 g	1840	$214,741	$272,743 ($232,247)	$501,846,932	2.80%	24.80%	79.80%	55.60%	5.40%	25.90%	1772	53 (26)	52
1250–1499 g	2433	$135,675	$180,346 ($180,388)	$438,780,665	1.80%	9.60%	63.50%	42.20%	3.10%	21.60%	2322	39 (23)	36
1500–1749g	3485	$88,189	$114,886 ($119,166)	$400,376,349	1%	3.60%	47.90%	14.50%	2%	17.40%	3354	27 (16)	25
1750–1999g	5410	$54,232	$75,293 ($100,897)	$407,337,737	0.60%	1.80%	33.10%	3.80%	1%	13.10%	5153	18 (12)	16
2000–2499g	16,483	$25,533	$42,998 ($90,127)	$708,735,398	0.40%	0.50%	20.90%	0.60%	0.50%	9.90%	15577	10 (10)	8
All low birthweight with specified BW	32,508	$48,906	$114,437 ($204,565)	$3,720,129,976	4.80%	7.60%	45.70%	13.90%	2.20%	20.20%	30953	23 (28)	14
Normal birthweight	727,538	$3,826	$6,743 ($30,360)	$4,905,759,132	0.10%	0%	0.40%	0%	0%	0.20%	525736	3 (4)	2

Though average expenditures for extremely immature infants < = 26 weeks) were high, as an age cohort, total spending on infants classified as moderately (33–34 weeks) and late (35–36 weeks) preterm (*n* = 40,473) was higher, with total expenditures for infants in these groups in excess of $1.46 billion, due to the larger number of newborns in these age strata. The infants who were the most preterm (<24 weeks, 24 weeks, 25–26 weeks, *n* = 1938) accounted for $868 million in healthcare services. Considerable variation in spending was also evident for infants within the same gestational age bands and birthweight groups, as can be seen from the cost distributions in Figs. 1 and 2, and through examination of the standard deviations in Table 1. A bimodal cost distribution is noted for both 35–36-week infants and 2000–2499 g infants.

### Association Between Adverse Events and Expenditures

We next analyzed the expenditures for neonatal complications associated with prematurity and low birthweight from birth through the first 6 months of life or until the time of infant death if occurring prior to 6 months (Table 2). Conditions included BPD, NEC, RDS, ROP, and sepsis (SEP). For preterm infants with a documented birthweight <2500 g and gestational age <37 weeks (*n* = 27,591), all adverse events were significantly associated with increased total spending (all *p* values <0.001) when included in the full model that also contained gestational age, birthweight, and sex. BPD was associated with the highest per infant expenditures with a more than double average multiplicative increase in 6-month spending (spending multiplier = 2.15) compared with infants without this complication. NEC also had a large association with increased spending, with an average multiplicative increase of 1.91. The remaining risk factors of RDS (spending multiplier = 1.66), ROP (cost multiplier = 1.16), and sepsis (spending multiplier = 1.29) had lower, but still substantial, associations with expenditures. Male sex had a statistically significant (*p* < 0.000001) spending multiplier of 1.08, indicating that expenditures for males are higher on average than expenditures for female preterm infants, even after adjustment for other factors.

**Table 2:** The effect of adverse events on expenditures.

Variable	Spending multiplier	95% confidence interval
*Adverse events*		
Bronchopulmonary dysplasia	2.15	(1.94, 2.38)
Necrotizing enterocolitis	1.91	(1.65, 2.22)
Respiratory distress syndrome	1.66	(1.58, 1.75)
Retinopathy of prematurity	1.16	(1.07, 1.25)
Neonatal sepsis	1.29	(1.22, 1.37)
*Effect of sex*		
Sex = Male	1.08	(1.03, 1.13)
*Birthweight*		
<500 g	1.43	(1.11, 1.85)
500–749 g	2.81	(2.27, 3.48)
750–999 g	2.25	(1.90, 2.67)
1000–1249 g	2.04	(1.78, 2.34)
1250–1499 g	1.90	(1.72, 2.11)
1500–1749g	1.66	(1.54, 1.80)
1750–1999g	1.34	(1.26, 1.43)
*Gestational age in weeks*		
<24	1.09	(0.84, 1.41)
24	2.46	(1.90, 2.67)
25–26	2.44	(2.00, 2.96)
27–28	2.61	(2.23, 3.06)
29–30	2.27	(2.02, 2.56)
31–32	2.16	(2.00, 2.33)
33–34	1.71	(1.62, 1.81)

Multivariable analysis of the impact of adverse event occurrence on 6-month expenditures for infants with a documented birthweight and gestational age (*n* = 27,591) in the claims cohort. All spending multipliers had *p* values <0.001, with the exception of gestational age <24 weeks (*p* = 0.53) and birthweight <500 g (*p* = 0.012). The spending multipliers in this table represent the full model which contained all risk factors, gestational age, birthweight, and gender as variables.

## Discussion

Our study provides estimates of healthcare expenditures through the first 6 months of life in the years 2008–2016 for newborns with billing codes indicating prematurity and low birthweight. Placing our study in context together with previous works [3–5, 20, 21] constructs a complex picture of the expenditures on prematurity and low birth with several important themes. Preterm infants are some of the most expensive patients in all of pediatrics [22], and there is an inverse relationship between gestational age, birthweight, [2, 23, 24] and infant costs. Our data suggest that both gestational age and birthweight provide similar predictors of neonatal mortality, prematurity-associated medical complications, hospital length of stay, and per individual medical expenditures.

This study adds to our understanding of healthcare spending for premature and low-birthweight infants in several important ways. Since the data are drawn from a nationwide commercial insurance database of paid claims, we have direct and comprehensive estimates for the complete third-party expenditures in this population. Claims data reflect the total expenditures on an individual and are not subject to certain limitations of cost measurement based on intermediate estimates such as hospital cost-to-charge ratios used in other analyses [3]. We are able to track the infants in our study regardless of where they received care (e.g. inpatient vs outpatient) and, as such, it expands on estimates based on hospital based-discharge data. Our study complements the existing literature which has focused on government insured patients (Medicare and Medicaid) or the patient population from a single state, and has not looked at this topic using a large national database of commercially insured patients. Although Medicaid covers nearly 50% of all US births [20], pricing and reimbursement dynamics in Medicaid may differ from commercial insurance. It is valuable to have both public and private spending estimates [25] by gestational age and birthweight bands in newborn populations to evaluate the financial impact of clinical or health policy interventions, particularly those targeted at specific gestational age or birthweight bands. For example, there is growing interest in structuring maternity bundled payment methodologies to include neonatal care costs but to exclude certain high cost neonates. The relative costs among different gestational age or birthweight cohorts could help guide the methodology and identify potential areas for focus in these arrangements.

The aim of cost-of-illness studies is to estimate as accurately as possible the true cost of production of services such as the treatment of prematurity. Hospitals measure, and report to government payers, their estimates of their own costs by converting their posted charges to costs using “cost to charge ratios” or similar cost allocation systems, and these systems are often used in Medicaid-based cost of illness studies. Although this approach provides a reasonable estimate, these approaches are weighted toward the overhead utilization of adult or older pediatric patients, and their applicability to neonates is uncertain. Conversely, payments by private insurers are affected not only by the true costs of production of medical care, but also by negotiated arrangements between institutions and payers, and this setting of payment rates typically involves factors other than the actual use of services by or illness acuity of the patient. Thus, neither the use of calculated costs from a Medicaid database, nor the large-scale reporting of expenditures of private payers used in the current study, will on their own reflect the cost experience of prematurity in the US. Estimates from each distinct population are necessary to understand the cost implications of policy recommendations or clinical interventions that impact prematurity and low-birthweight infants, particularly in the current context, when the relative merits and weighting of private versus public payment for medical care is being debated. It is reassuring that, although the absolute dollar amounts may vary (with private-payer estimates typically being higher), the estimates reported here are in fact fairly similar in magnitude to those in recent Medicaid-based analyses. Moreover, the patterns of costs—for example, the variation by gestational age or illness acuity—are similar across different populations and methodologies.

While the least mature infants have the highest average per individual medical expenditures, more mature infants as a cohort, have higher total expenditures. Infants born at 33–36 weeks gestation represent approximately 80% (*n* = 40,473) of all preterm births and 38% of expenditures (expenditure = $1.46 billion) among all preterm infants. By contrast, infants born at less than = 26 weeks represent ~4% of all preterm infants and 22.5% of expenditures (expenditure = $868 million). Given the magnitude of expenditures in older preterm infants, improvements in healthcare delivery of infants in this gestational age band represent an important opportunity to improve the aggregated economic and clinical burden of prematurity.

One interesting observation from our analysis is the appearance of bimodal distributions of expenditures for some gestational age and birthweight groups. This phenomenon, to our knowledge, has not been widely reported. This pattern is most obvious in the <24 week and <500 g infants (Figs. 1 and 2, who had an average expenditure of $242,887 and $247,994, respectively, but median expenditures of $7318 and $6379. The source of this bimodality is due to the high mortality rates in these groups, as infants who do not survive will receive fewer services and have lower expenditures. This finding has been previously observed in prior studies that compare hospitalization costs between survivors and non-survivors in these groups [6, 26]. The pattern is also prominent in near term infants born at 35–36 weeks and 2000–2499 g infants, though it is unlikely that mortality explains the trend in these more mature groups. For example, in the graph of 35–36-week infants, one mode aligns nearly perfectly with full-term infants [27], while the other mode appears to align with the 33–34-week infants. The same appears to be true for the infants born weighing 2000–2499 g.

Incorrect assignment of gestational age and birthweight could influence the shape of the cost distributions, though it is unlikely that this is systemic enough to result in pronounced separation of modes for infants born at 35–36 weeks, but not affect successive gestational age bands in a similar manner. One explanation is that the much higher rate of planned delivery of infants with congenital anomalies or pregnancy complications at this gestational age band explains for this apparent bimodality. Another explanation for this trend may be the influence of non-clinical “supply sensitive” care component described in other studies, where infants who could be managed in less acute neonatal care settings are monitored in the NICU [7]. Though answering this question is beyond the scope of this work, the cost data imply there are in fact two types of infant (high acuity/low acuity) who are born on the cusp of full term/normal birthweight. Precise characterization of such subgroups may lead the way for more informed and efficient delivery of care.

## Limitations

Several limitations should be acknowledged. This study examines a commercially insured population, and thus may not be reflective of the natural history or adverse consequences of prematurity in the US population as a whole. In addition, this study population may be subject to different pricing dynamics than those with government-based insurance, such as Medicaid, which covers nearly 50% of all US births [20]. While Medicaid does cover many deliveries, the amount paid is a derivative of the payments made by commercial insurance arrangements and while the absolute dollars may vary from our data, the relationships and observations seen here should hold true for Medicaid as well. While caution is needed in applying these estimates to predominantly Medicaid populations, similarly cost estimates from Medicaid populations cannot be generalized to commercially insured populations. Estimates from each distinct population are necessary to understand the cost implications of policy recommendations or clinical interventions that impact prematurity and low-birthweight infants.

A second limitation is that claims data may be incomplete, and diagnoses derived from billing codes may not be indicative of patients’ true gestational age, weight, or clinical condition. ICD-9/10-CM codes were not intended to capture all infants born preterm or with low birthweight, only the subset who require medical care due to complications of prematurity or intrauterine growth retardation [5]. Thus, the expenditures reported here may be an over-estimate or represent an upper bound on the true cost for these infants for the first 6 months of life. In addition, our definitions of “full term” and “normal birthweight” may include infants who were in fact preterm and/or low birthweight if they did not receive a code for these conditions.

Our estimates do not include outpatient pharmacy costs because we did not have those data for everyone in the cohort, nor do they include non-covered parental out-of-pocket expenditures. Finally, these are estimates of the short term (6 months) costs of prematurity and low birthweight. The consequences of preterm and low-birthweight infants frequently follow an individual through childhood or early adulthood.

## Conclusions

Healthcare spending continues to be a complex and multifaceted issue in the United States and the cost of prematurity is of broad interest to patients, providers, payers, and policy analysts. This analysis has multiple strengths including large cohort size, distribution of births (all states except California), granularity of costs by gestational and birthweight strata, and direct expenditure data. Accurate understanding of healthcare expenditures is necessary to guide research priorities, investments in intervention strategies and to address variations in the cost of care. These data suggest that efforts that target moderately preterm infants may have a larger impact on reducing the overall clinical and economic burden of prematurity relative to efforts which concentrate on infants that are born earlier.

## Supplementary information

Supplementary Material
