# Supplementary material for: Estimates of healthcare spending for preterm and low-birthweight infants in a commercially insured population: 2008–2016
Source: J Perinatol. 2020 Feb 26;40(7):1091–9. doi: 10.1038/s41372-020-0635-z (PMC7314662; doi:10.1038/s41372-020-0635-z)
Supplement: Supplementary file 1 — Supplementary Material [file 41372_2020_635_MOESM1_ESM.docx]

**Supplemental Figure 1**

We compared the distribution of infants born in each gestational age group to nationally representative figures provided by the CDC (Table 23 on Page 58: https://www.cdc.gov/nchs/data/nvsr/nvsr66/nvsr66_01.pdf). In particular, the CDC provides the number of infants born at < 28 weeks, 28 – 31 weeks, 32-33 weeks, and 34-36weeks. Because these groups do not align perfectly with the groups defined by ICD codes, we adopted to following strategy for comparison.

The following figure shows the cumulative fraction of preterm infants born at or below the gestational age displayed on the x-axis in both our data and the numbers provided by the CDC. For example, in our data (red line) just over 10% of preterm infants were born at or below 30 weeks of gestation. Likewise, approximately 16% of infants were born at or below 31 weeks of gestation according to the CDC data.

Points on the graph indicate where the groups occurred and are connected by lines to aid in visualization, though the lines are not interpolations between the points.

As can be seen in the graph, the fraction of infants born at or below each gestational age group in our data agrees strongly with the estimates provided by the CDC, increasing the generalizability of the findings in the main text.

**Supplemental Figure 1:** Comparison of birth rates at different gestational age in the Aetna population and national rates provided by the CDC. The x-axis is gestational age at time of birth and the y-axis is the cumulative fraction of infants born at that gestational age.

**Assessment Gestational Age and Birth Weight Accuracies**

To assess how accurate birth weight and gestation age are captured by ICD codes, we estimated the number of infants who had a clinically implausible birth weight and gestational age combination. A birth weight was determined to be implausibly *low* if it was 3 standard deviations below the mean birth weight reported in Olsen et al (Olsen *et al.*, 2010), using the infant’s minimum possible gestational age, as the database contained only ranges for gestational age and birth weight rather than precise values. For example, if the infant received a code indicating 31-32 weeks (ICD-9 765.26), we used three standard deviations below the mean for 31-week infants from Olsen et al as the cutoff point. A corresponding set of analyses was used to determine infants who had an implausibly *high* birth weight using the infant’s maximum possible gestational age.

Overall, of the preterm infants with documentation for both gestational age and birth weight, 0.27% had implausible combinations. The highest percentage of implausible combinations occurred in the 24-week gestational age band, where 2.9% of infants were identified as outliers. Because these rates were low we conclude both measures are captured with high fidelity in the data.

**References**

Olsen, I. E. *et al.* (2010) ‘New intrauterine growth curves based on United States data.’, *Pediatrics*, 125(2), pp. e214-24. doi: 10.1542/peds.2009-0913.
